# Cholesterol dependent cytolysins and the brain: Revealing a potential therapeutic avenue for bacterial meningitis

**DOI:** 10.3934/microbiol.2023033

**Published:** 2023-08-21

**Authors:** Tjokorda Istri Pramitasuri, Ni Made Susilawathi, Ni Made Adi Tarini, AA Raka Sudewi, Matthew C Evans

**Affiliations:** 1 Doctoral Program in Medical Science, Faculty of Medicine, Universitas Udayana, Bali, Indonesia; 2 Postgraduate Research Student, Faculty of Medicine, Imperial College London, United Kingdom; 3 Department of Neurology, Faculty of Medicine, Universitas Udayana, Bali, Indonesia; 4 Department of Microbiology, Faculty of Medicine, Universitas Udayana-Rumah Sakit Umum Pusat Prof Dr dr IGNG Ngoerah, Bali, Indonesia; 5 Pain Research, Department of Surgery and Cancer, Faculty of Medicine, Imperial College London, United Kingdom; 6 Department of Brain Sciences, Care Research and Technology Centre, UK Dementia Research Institute, London, United Kingdom

**Keywords:** cholesterol dependent cytolysin, bacteria, meningitis, central nervous system, brain

## Abstract

Bacterial meningitis is a catastrophic nervous system disorder with high mortality and wide range of morbidities. Some of the meningitis-causing bacteria occupy cholesterol dependent cytolysins (CDCs) to increase their pathogenicity and arrange immune-evasion strategy. Studies have observed that the relationship between CDCs and pathogenicity in these meningitides is complex and involves interactions between CDC, blood-brain barrier (BBB), glial cells and neurons. In BBB, these CDCs acts on capillary endothelium, tight junction (TJ) proteins and neurovascular unit (NVU). CDCs also observed to elicit intriguing effects on brain inflammation which involves microglia and astrocyte activations, along with neuronal damage as the end-point of pathological pathways in bacterial meningitis. As some studies mentioned potential advantage of CDC-targeted therapeutic mechanisms to combat CNS infections, it might be a fruitful avenue to deepen our understanding of CDC as a candidate for adjuvant therapy to combat bacterial meningitis.

## 1. Introduction

Bacterial meningitis is a severe infection of the central nervous system (CNS) associated with high mortality and long-term neurological sequelae in survivors [1]–[3]. A range of incidence data have been reported depending on the setting, with higher incidences reported in Sub-Saharan Africa, the so-called African meningitis belt (10–40 in every 100,000 people), than in United States and European countries (between 0.7 and 7.1 per 100,000 people) [4]–[7]. It was said to be the cause of approximately 2% of deaths in children, who also have a five-times increased in risk of developing neurodevelopmental impairments (NDIs) [8],[9]. Some of the epidemiological variation in bacterial meningitis will be related to the specific pathogens involved, with their own region-dependent distribution, which is further impacted by sociocultural factors. For example, regions with higher consumption of raw pork like South East Asia will have higher prevalence of *Streptococcus suis* meningitis [10]. With regards to severity (e.g., mortality rate, long-term outcomes), bacterial meningitis is more severe than viral, fungal or aseptic forms of meningitis [1]–[4]. This has led researchers to question what is inherently different for bacteria compared with other micro-organisms in terms of the effect on the CNS that results in this more aggressive course.

Our understanding of the pathogenesis of bacterial meningitis is still incomplete. However, deepening the understanding of disease mechanisms of disease could lead to more targeted, organism-specific therapies for these conditions [11]. What has emerged is that various micro-organisms seem to share one particular virulence factor, known as pore forming toxins (PFT). The PFTs are the largest category of bacterial virulence factors in bacterial meningitis. These PFTs localize to the bacterial cell surface and can be either secreted or released during bacterial autolysis to induce cytotoxic effects by perforating the plasma and intracellular organelle membranes of host cells [12],[13]. Among them, the largest sub-family of PFTs to have been studied so far as the cholesterol dependent cytolysins (CDCs).

The CDCs are secreted by various genera of gram-positive bacteria such as *Streptococcus* and *Listeria*. Examples of CDCs include pneumolysin (PLY) from *Streptococcus pneumoniae* [14],[15], suilysin (SLY) from *Streptococcus suis* [16], β-hemolysin/cytolysin toxin (β h/c) from Group B *Streptococcus* [17], streptolysin O (SLO) from *Streptococcus pyogenes* [18] and lysteriolysin O (LLO) from *Lysteria monocytogenes* [19]. The relationship between CDCs and pathogenicity in these meningitides is complex and involves interactions between CDC, blood-brain barrier, glial cells and neurons [20]–[24].

This review highlights and delineates mechanisms by which CDC interacts with different brain cells to disrupt the normal CNS environment, with a particular emphasis on neuroinflammatory signaling on brain barrier, glial cells and neurons. A better understanding of these pathological mechanisms will elevate our reasonings to utilize CDC as a future therapeutic target of bacterial meningitis.

## 2. Cholesterol dependent cytolysin in bacterial meningitis

There are various methods used by bacteria to enter the nervous system to cause damage, much of which focuses on disrupting the permeability of the blood-brain barrier (BBB). Once they gain access to the cerebrospinal fluid (CSF) bacteria multiple and bacteria release pathogenic factors including cytolysin, which induces a strong inflammatory response inside the brain microenvironment, affecting the meninges and glial and neuronal cells. CDCs are one family of these virulence factors, which result in lysis of affected cells in a cholesterol-dependent fashion. According to various studies, CDCs have been implicated in brain barrier disruption, brain inflammation (including glial cell activation and a surge of inflammatory chemicals) and neuronal injury as end outcomes of the pathways. Table 1 illustrates some of the common types of CDCs associated with various bacterial meningitis organisms and demonstrates and compares some of their characteristic in terms of the cell types they have been demonstrated to effect.

**Table 1.** Studies Assessing the Role of CDCs in Meningitis

CDC	Type Of Study	Cell Lines / Animal Model Used	Brain Barriers Dysfunction	Brain Inflammation	Neuronal Damage	References
PLY	*In-vitro*	Primary mouse and rat astrocytes, mixed mouse glial cell cultures, human neuroblastoma cells, primary rat cortical neurons	√	√	√	[14],[15],[21],[25]–[35]
	*In-vivo*	Mice, rats	√	√	√	[21],[28],[30],[31],[34],[36]
SLY	*In-vitro*	BMEC, primary murine astrocytes	√	√		[37]–[41]
	*In-vivo*	Mice, piglets	√	√	√	[42],[43]
β-h/c	*In-vivo*	Mice	√			[17],[44]
SLO	*In-vitro*	BMEC	√		√	[18],[45]–[47]
LLO	*In-vivo*	Mice		√		[48]

Cholesterol-rich membrane microdomains or lipid rafts are the integral component in the pathogenesis. They are considered as the initial place for CDCs to interact with the cells. Lipid rafts have outward looking receptors at their surfaces as the binding site of pathogens and endocytosis machinery which utilized to guide the microbe to enter the host cells [49]. The CDCs bind with cholesterol membrane by utilizing their highly conserved tryptophan (TRP)-rich motif in domain 4 (D4) of their 3-dimensional (3D) protein structure [50],[51].

## 3. Blood-brain barrier dysfunction

The blood-brain barrier (BBB) is an integral structure delineating the CNS from the rest of the body and key in maintaining the unique immunological environment in the CNS. The BBB is made up of brain microvascular endothelial cells (BMECs) and periendothelial structures such as pericytes, astrocytes and basal membrane which surrounds cerebral microvessels [52]. One of the roles of the BBB is to protect the CNS from invading pathogens.Therefore, breakdown of this structure leaves the CNS vulnerable to invasion by infectious agents causing, among other things, infectious meningitis. Various mechanisms seem to play a part and even foster in this disruption of the BBB, including endothelial activation by various chemical mediators or signaling mechanisms and alteration on structural organization of its neurovascular unit (NVU) [20],[53].

### 3.1. Effect on The Capillary Endothelium

After bacterial invasion and replication in the brain environment, host pattern recognition receptors (PRRs) recognize pathogen-associated molecular patterns (PAMPs), including bacterial toxins [54], leading to activation of pro-inflammatory cytokine pathways, particularly from microglia and astrocytes [55],[56]. Then, this acts on the nearby capillaries, activating the endothelial cells, resulting in upregulation of molecules such as intercellular adhesion molecule-1 (ICAM-1), vascular cellular adhesion molecule-1 (VCAM-1) and selectins, which allows selective recruitment of leukocyte populations [57]. Moreover, pro-inflammatory events will trigger alteration of tight junction (TJ) proteins, which more globally affect vascular permeability [52],[57],[58]. CDCs have been demonstrated to manipulate each of these pathways.

Immune cell recruitment into the CNS, especially influx of leukocytes into the subarachnoid space is a pivotal event in bacterial meningitis, and indeed demonstrating specific leukocyte populations in the CSF is an early diagnostic marker. Leukocyte influx-induced brain injury is mediated by elevation of reactive oxygen metabolites, pro-inflammatory cytokines and proteolytic enzymes [59]. Leukocytes have on their cell surface integrin ligands (e.g., LFA-1, VLA-4) or sialylated carbohydrates (e.g., sialyl Lewis X), which bind to adhesion molecules such as ICAM-1, VCAM-1 or the selectins and this mechanism mediates leucocyte rolling, firm adhesion and subsequent extravasation into the CNS [60]–[62]. If the transport of leucocytes into extracellular chamber exceeds its physiologic range, tissue injury can occur, including damage to brain capillary endothelial cells [60].

Doran (2003) demonstrated that β-h/c, a CDC toxin from Group B *Streptococcus* (GBS) was able to provoke BBB activation through inducing interleukin (IL)-8 release *in-vitro*, which increased the CNS bacterial count in mice. The study also showed a marked reduction in the expression of the chemokines Groα and Groβ after infection of a GBS strain lacking β-h/c [17]. In conjunction with this, a number of CDCs including PLY and SLY induce morphological changes to BMECs, including dilation of endoplasmic reticulum, chromatin clumping, reduction of cytoplasmic density and elevation of lactate dehydrogenase [35],[37],[44]. In another study, stimulation of THP-1 monocytes by purified SLY induced expression of ICAM-1, CD11a/CD18, and CD11c/C18 at low doses (1 and 0.5 µg/mL). On the contrary, even high dose (200 µg/mL) of capsular polysaccharide (CPS) did not upregulate all the three molecules, demonstrating some specificity in activation of these pathways [60].

Neutrophil extracellular traps (NETs) are an interesting structure composed of deoxyribonucleic acid (DNA), histones and proteolytic enzymes, which are released by neutrophils after activation in infection and have a role in binding and killing pathogens separate from the neutrophils other role in phagocytosis [63]. The significance of NETs has been demonstrated in various infections, including most recently SARS-CoV-2, but also in non-infectious conditions such as ischemic stroke [64]. As the field of NETs develops there is increasing evidence of their role in bacterial meningitis. For example PLY causes NET formation *in-vitro*, independent from TLR4 and reactive oxygen species (ROS) production [65] and SLY has also been demonstrated to trigger NET formation [66]. There is a complex bidirectional relationship between neutrophils and platelets in infection, whereby activated platelets lead to NET production. NETs in turn can enhance platelet activation [67],[68], which may in part explain the thrombotic complications that can occur in infection. However, other pathways than NETs are involved in this. For example, one study demonstrated that treatment in vitro with PLY induced production of extracellular vesicles (EVs) from both neutrophils and platelets and that neutrophil derived EVs are capable of inducing platelet activation [69].

The BMECs are joined by tight junctions (TJs), which is an integral component to the permeability of the [70], and any disruption to BMEC can contribute to disease mechanisms. CDCs are capable of disrupting the integrity of the BBB via a number of mechanisms, including creation of membrane pores, but additionally via triggering pro-inflammatory cytokine cascades that have a secondary effect of BBB permeability via altering TJs structure [20],[53]. The TJs in BMECs consist of occludins, claudins, and adhesion molecules which are linked into the actin cytoskeleton through zonula occludin (ZO) and cingulin [57],[58]. Whilst these mechanisms require further elucidation, it is clear there is an important interaction between CDCs, proinflammatory cytokine/chemokine pathways, and tight junction proteins.

A study by Sui *et al*. (2022) has shown that SLY from *Streptococcus suis* induced cerebral microvascular endothelial cells (CMEC) to release tumor necrosis factor (TNF), which resulted in increased microvascular permeability, which appeared to be dependent on secretion of phospholipase A2. The mechanistic role of TNF was further highlighted by the demonstration that the TNF inhibitor pomalidomide blocked the effect of SLY on BBB permeability [42]. PLY has also known to induce the release of TNF in numerous cell types including brain endothelial cells [35],[71],[72]. SLY has its undeniable role as a potent TNF inducer in other cells such as monocytes and mast cells by inducing p38 mitogen activated protein kinase (MAPK) and protein kinase-C (PKC) dependent pathways [73],[74].

The exact mechanisms of the effect of TNF on the BBB are complex, and involve numerous subsidiary pathways including hypoxia inducible factor-1alpha (HIF-1alpha)/vascular endothelial growth factor (VEGF)/VEGF receptor-2 (VEGFR-2)/extracellular regulated protein kinases (ERK) pathways, and the end result of these pathways is to downregulate or otherwise disrupt tight junction proteins that are integral for BBB integrity [42],[75]–[79]. After being released from the brain endothelial cells, TNF induces HIF-1a activation, which, in turn, triggers the activation of ERK by VEGF, which leads to loss of expression of various tight junction proteins including occludin and ZO-1, or changes to their phosphorylation status [77],[79]. As the essential component of tight junction proteins in BBB, occludin degradation has been demonstrated to be a primary factor underlying increased BBB permeability in various studies [80]–[82]. As alluded to above, another important step in TNF-induced permeability changes in the expression of the group III secretory phospholipase A2 (PLA2G3). Sui *et al*. (2022) has demonstrated that after SLY-induced TNF expression, PLA2G3 is released which resulted in the increase of paracellular permeability of human cerebral microvascular endothelial cells (hCMEC) both *in-vivo* and *in-vitro* [42].

### 3.2. Remodelling of NVU

The blood-brain barrier, though, is more than simply a collection of endothelial cells joined by tight junction proteins. The functional anatomy of the blood brain barrier additionally involves astrocyte end-feet, pericytes, myocytes and connective tissue proteins, all intimately related to the neighbouring neurons, to provide nutritional supply and transfer integral for neuronal function [20],[83]. This is collectively referred to as the neurovascular unit (NVU) and if any part of this unit doesn't perform its function, the BBB becomes compromised. PLY was the first CDC demonstrated to be capable of inducing remodelling of the endothelial cell actin cytoskeleton, thereby disrupting the NVU. This remodelling mechanism relies on small GTPases and acts in a cholesterol-dependent manner [27],[35]. A decade later SLY was also found able to alter the actin cytoskeleton of human brain microvascular endothelial cells (hBMEC) via a RhoA-GTPase dependent pathway [38]. As was the case for PLY, the SLY-induced activation of RhoA was dependent on the concentration of cholesterol, supporting the idea that the cholesterol-dependent characteristics of many of these CDCs applies to broader mechanisms than just the pore-forming mechanism [38],[41].

Downstream of RhoA signalling, the molecular mechanisms involved in SLY-induced cytoskeleton remodelling are complex, involving a number of pathways including ROCK, citron kinase, and Drosophila homologue of mammalian diaphanous related protein (mDia1). ROCK then phosphorylates and inhibits myosin light chain (MLCK) and activates LIM kinase. Inactivated MLCK increases the level of p-MLC as the positive regulator of acto-myosin network, whilst LIMK inactivates cofilin [84],[85]. The endpoints of these signalling pathways are increased levels of p-MLC and p-cofilin which lead to the formation of actin stress fibres. The effect of RhoA on citron kinase results in actin filament bundling during cytokinesis and facilitates cell separation [86]. Separately, CDC-induced activation of RhoA activates mDia1 and leads to the de novo actin polymerization [87],[88].

**Figure 1.**
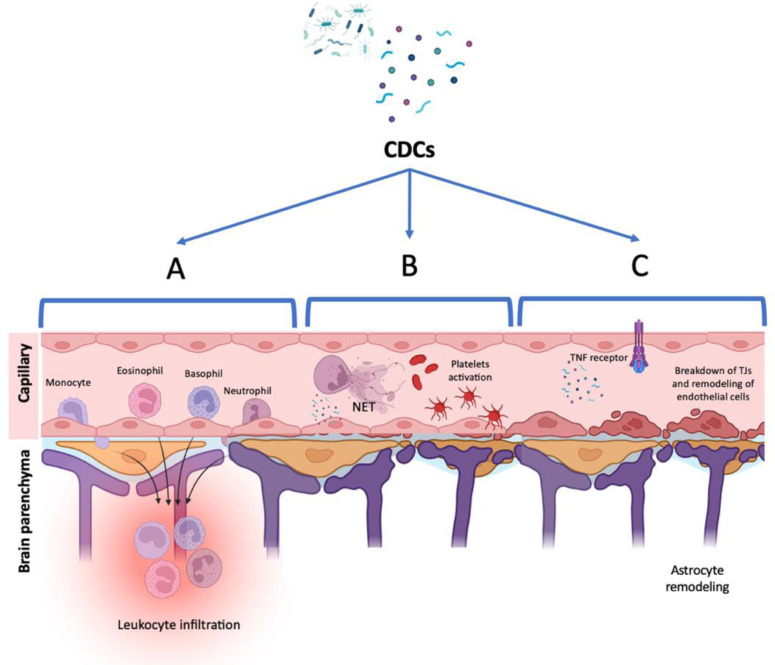
Effect of CDCs on BBB. As the aftermaths of CDC release after bacterial invasion, the toxins have been demonstrated to manipulate various pathological pathways including A) Leukocyte influx, B) activation of NETs, C) TJ breakdown, BBB leakages and astrocyte remodeling. Figure created in Biorender.com.

A study by Fortsch (2011) has revealed the capacity of PLY to cause astrocyte actin cytoskeletal remodelling *in-vitro*. At high concentrations PLY produces cell lysis via the pore-forming mechanism, but when added a sub-lytic concentration to primary astrocyte cultures, they demonstrated morphological changes caused by actin depolymerization. Interestingly this mechanism was independent of sodium and calcium influx, but rather seemed to be dependent on the lytic, pore-forming mechanism, even though PLY was applied at sublytic concentrations. The authors demonstrated that when using the non-lytic PLY mutants W433F-pneumolysin and delta6-pneumolysin (which disrupt the pore-forming mechanism), that the effect on astrocyte shape changes and retraction was abolished. Whilst this has yet to be demonstrated *in vivo*, it seems plausible that retraction of astrocyte end feet would interfere with the structural integrity of the NVU, resulting in increased BBB permeability and increased ability of systemic organisms to cross the BBB [25]. A summary on these mechanisms can be seen on Figure 1. On the other hand, interactions between CDCs and other supporting cells of the NVU such as the pericyte needs better characterization in future studies [89].

## 4. Brain inflammation

In bacterial meningitis, organisms gain entry to the body, and initially enter the systemic blood supply resulting in bacteraemia with or without a septic response, and due to some of the mechanisms described above, are then able to cross a compromised BBB to enter the CSF, from which they are able to cause often catastrophic neuronal injury. Along with bacteria crossing the BBB, due to both central and systemic cytokine/chemokine signalling, leukocytes also enter the CNS, triggering pro-inflammatory signalling cascades, which can also contribute to neuronal damage. Once bacteria are in the CNS, CDCs produced from these bacteria are an important virulence factor, affecting both glial cells (astrocytes and microglia) and neurons. Glial cell activation is regulated by various distinct molecular pathways, but the nuclear factor kappa B (NF-kB) is generally acknowledged as the primary pathway driving these morphological and physiological changes [90],[91].

### 4.1. CDC and microglia

Microglia are the specialised macrophage population of the brain and are capable of cytokine/chemokine production, production of reactive oxygen species (ROS) and phagocytosis all of which can exacerbate neuronal injury, giving a worse prognosis for patients with bacterial meningitis [92],[93].

Studies have shown that CDC production is an important part of bacteria-induced microglial activation associated with various pathogens, both from work focused on creating ΔCDC mutants and that with purified CDCs from bacterial isolates. The activation of microglia after infection has known to happen after their pattern recognition receptors (PRRs), e.g., toll-like receptors (TLRs), sensing large pathogen-associated molecular patterns (PAMPs) such as lipopolysaccharide (LPS) and CDCs. PLY [71],[94],[95], SLY [16],[96],[97], LLO and SSO [98] are potent stimulators of TLR4, which is highly expressed on microglia, astrocytes and brain neurons [99]–[101].

The CDC-induced TLR-4 signalling mechanism can occur via two pathways, either MyD88 or the Toll/interleukin-1 receptor- (TIR-) domain-containing adaptor inducing interferon-B (TRIF). First, TLR-4 associates with its extracellular binding partner, myeloid differentiation factor 2 (MD-2), resulted in TLR4-MD-2 complex. This complex binds with the CDC and recruits another similar complex to produce a homodimer. Through its ability of transmitting intracellular signals, the TLR4-MD2 recruits toll-interleukin-1 receptor (TIR) domain-containing adaptor protein (TIRAP) and myeloid differentiation primary response 88 (MyD88). The MyD88 then interacts with Interleukin-1 receptor-associated kinase 4 (IRAK4) death domain. This series of events recruits tumor necrosis factor receptor-associated factor (TRAF)-6, which then, together with Ubc13 and Uev1A, initiate the activation of transforming growth factor-β-activated kinase 1 (TAK)1 and TAK binding protein (TAB)1-TAB2/3 complex. This event further activates a complex consisting of inhibitor of nuclear factor-κB (IκB) kinase (IKK)-a, IKK-b and IKK-y which trigger the entry of NF-kB into the nucleus, resulting in transcription of messenger ribonucleic acid (mRNA) for proinflammatory cytokines such as interleukin (IL)-1B, IL-6 and TNF. The MyD88 pathway also activates mitogen activated protein kinase (MAPK) signalling pathway, which causes p38, ERK and janus kinase (JNK) to accumulatively trigger AP-1 translocation into the nucleus. On the other hand, TLR4-MD2 causes TRIF-related adapter molecule (TRAM) to translocate to the cytoplasm, which activates TRIF-dependent pathways, leading to activation of the downstream TBK1/IKK complex and TRAF3, in turn causing phosphorylation of interferon regulatory factors (IRF)3 and IRF7. The end result of these signal pathways is an elevation of type 1 IFN (IFN-alpha and IFN-ß) gene expressions. The IFN-alpha has known to increase the expression of pro- markers of a pro-inflammatory microglial phenotype (MHC-II and CD86) in BALB/c mice [102]. After their activation by CDCs, morphological changes [95], increased nitric oxide production and other inflammatory mediators such as TNF, IL-1b were observed [95].

Other than TLR4-dependent responses, there are various ways taken by CDCs to activate microglia such as interaction with infiltrating leukocytes and upregulation of microRNA (miR)-155. Zhang *et al*. (2018) showed that virulent *Listeria monocytogenes (L. monocytogenes)* strain caused upregulation of miR-155 in whole brain and significantly induced brain leukocyte influxes in mice which led to microglial activation, whereas infection with ΔLLO mutants did not [48]. The CDC-induced microglial activation also suggested to be a result from the ability of CDCs to bind with glycans. Major members of CDCs; PLY, SLY, LLO and SLO have high-affinity lectin activity that recognizes glycans as their cellular receptors [103]. Glycans are polysaccharides, serving as a fundamental structural component of the cells in all living organisms. In the CNS, glycans play pivotal roles in homeostasis and immune cells activation, including glial scar formation. A recent study which investigates the role of glycome in neuronal inflammation has proven that during glial activation, glycans are significantly expressed and essential for microglial function [56].

In addition to microglia activation, the pathogen-induced microglia pyroptosis has been known to significantly contribute to neuroinflammation and neuronal damage due to its ability to induce release of intracellular inflammatory mediators. A study revealed that PLY induced microglia pyroptosis in a caspase-1 and interleukin (IL)-1 dependent manner [24].

### 4.2. CDC and astrocytes

The physiological role of astrocytes is important for the homeostasis of CNS environment. Astrocytes have a role in nutritional transport from blood circulation to neurons, metabolism of chemical mediators, synaptic transmission, synaptic activity and neurovascular coupling. Some alterations in factors associated with aforementioned physiological mechanisms also trigger the release of cytokine, reactive oxygen species (ROS), reactive nitrogen species (RNS) and alter glutamate-glutamine metabolic flux, which accumulatively exacerbate the inflammatory state [104]–[109]. Moreover, chronic stimulation of astrocytes impedes axonal regeneration and level of neurotrophic proteins needed for maintaining normal neurophysiological functions [23],[110],[111]. Astrocyte activation can be regulated by various factors released from activated microglia or injured neurons, resulting in a complex inter-cellular relationships through the course of an infectious disease of the CNS [112].

CDCs have been identified as the likely causative factors in neurotoxic astrogliosis in some recent studies. Wippel *et al*. (2013) has observed that after exposure to non-lytic concentration of PLY there is an increase of calcium influx and subsequent glutamate secretion on mouse astrocytes cell line, followed by permanent dendritic swelling, loss of dendritic spine and synaptic loss via activation of n-methyl d-aspartate receptors (NMDAR) [33]. This mechanism was previously observed in ischemic brain disorders where NMDA activation leads to Ca2+ and Na2+ overload in post-synaptic neurons, resulting in excitotoxicity cell death [113].

## 5. Neuronal damage

Neuronal damage is a primary concern in infectious disease of CNS, and even when not fatal can lead to significant morbidity including cognitive impairment and movement disorders [31],[114] and can be said to be the final common pathway various pathological mechanisms in bacterial meningitis [34],[114],[115]. To date, the only CDC which has been established to have a catastrophic effect on neuronal life cycle is PLY. PLY acts as a key factor in modulating pneumococcal interactions with neurons. Purified PLY binds with the neuronal plasma membrane through its Trp-rich motif amino acid sequence. Then, binding occurs via interaction between the pilus-1 adhesin RrgA with β-actin, which is damaging to neurons due to elevated intracellular Ca2+ concentration in the mitochondria of neurons and disassembly of actin cytoskeleton [34].

Mitochondrial damage has been identified as a crucial factor in the death of neuronal cells [115]. Two studies revealed the role of PLY in inducing neuronal cell apoptosis via mitochondrial damage in primary rat hippocampal and cortical neurons. This process was independent of caspase signalling [14],[36]. In primary rat hippocampal neurons, PLY induced a rapid loading of calcium (Ca^2+^) into mitochondria. This induced permeability transition pore (PTP) dysfunction, leading to neuronal cell death [116],[117]. Similar neuronal toxicity was seen for SLO, which did not require internalization of *Streptococcus pyogenes*, indicating that CDCs act independently [46].

Physiologically, Ca2+ concentration in mitochondria is controlled by an electrogenic Ca2+ uniporter (MCU) and voltage dependent anion channel (VDAC), which collectively lead to accumulation of Ca2+ in the mitochondrial matrix. A physiologic balance in influx-efflux of Ca2+ is achieved by well-functioning Na+/Ca2+ and H+/Ca2+ exchangers along with PTP. However, if an event (e.g., infection) triggers activation of apoptotic signals, these signals along with Ca2+-mediated cellular signals will induce long-lasting openings of PTP, causing swelling of mitochondria, followed by accumulation of caspase cofactors into the cytosol which led into the beginning of cell death process [117],[118].

The aforementioned effect of PLY on astrocytes has opened a new insight in astrogliosis-induced neuronal damage. PLY has been shown to independently boost local glutamate release, activate NMDAR and induce persistent dendritic abnormalities through its ability to activate astrocytes. Dendritic focal swellings, also known as beads or varicosities, are one of two early hallmarks of neuronal toxicity, along with mitochondrial failure [119].

**Figure 2.**
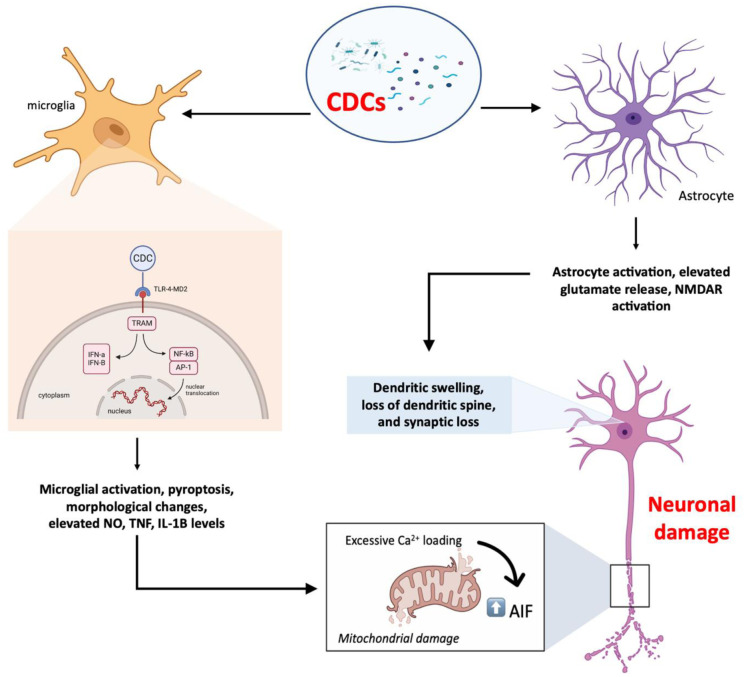
Mechanisms on CDCs-induced brain inflammation and neuronal damage. Following CDC activation of glial cells, various catastrophic downstream signaling pathways are activated. They continue to trigger the two early hallmarks of neurotoxicity, dendritic modification and mitochondrial damage, resulting in neuronal death. Figure created in Biorender.com.

PLY has been demonstrated to cause mitochondrial dysfunction and subsequent release of apoptosis inducible factor (AIF) as the final stage of neuronal apoptosis [36]. The mitochondrial AIF has been established in various studies as the main mediator of caspase-independent apoptosis-like neuronal cell death [120],[121]. This release of AIF was mediated by excessive intracellular Ca2+. In the same study, this *in-vitro* effect was tested in a rabbit model of pneumococcal meningitis *in-vivo*. High level of PLY was found in dentate gyrus area of hippocampus, particularly in the area of dying neurons, by using immunohistochemical staining with specialized anti-PLY antibody and peroxidase-conjugated secondary antibody [36]. The mechanisms involving brain inflammation and neuronal damage is summarized in Figure 2.

## 6. CDC-based therapeutic approach: potential future adjuvant treatment?

Multifaceted roles of CDC in CNS cells increase its possibility of being a future candidate of adjunctive treatment to combat bacterial meningitis or using it as a preventive approach by designing CDC-based vaccine. Current progress of CDC-based treatment strategy relies on the development of several efforts to induce neutralizing antibodies. A study by Salha *et al*. (2012) explored that a novel detoxified pneumolysin derivative (PlyD1), which is a genetically modified protein, and has been demonstrated capable to induce neutralizing antibodies against PLY. The study also indicated that the aforementioned antibody-mediated protection resulted in an excellent protection against lung injury and lethal intranasal challenge with pneumococci [122]. Thought this study was not specifically investigation CNS infection, it nonetheless highlights the potential advantage of this as a therapeutic approach. Neutralizing antibodies against SLY [123], SLO [47] and LLO [124]–[126] have been studied in the last two decades, but more studies are needed to confirm their effects on CNS cells during meningitis. However, antibody-based therapy approaches typically only work for a given toxin in a specific manner, making it a challenge to overcome the large variability of toxins [127].

An interesting finding came from a study by Subramanian *et al*. (2020) which reported an *in-silico* based approach to design a mannose receptor (MRC-1) derived peptide, to examine its effect on CDC-induced cytolysis *in-vitro* and assessed its effect on PLY-induced epithelial barrier damage in a 3D lung tissue model. The MRC-1 peptides successfully countered the deleterious effects of PLY, SLO and LLO by blocking cytolysis, production of pro-inflammatory cytokines, bacterial uptake and intracellular bacterial survival. These studies demonstrate the potential advantage of CDC-targeted therapeutic mechanisms to combat CNS infections [128].

A number of natural substances counteract on CDC and highlight some promising results. Myricetin, a flavonoid compound isolated from bayberries, has recently been proven as a potent inhibitor of SLY through molecular modelling, biological assays, *in-vitro* and *in-vivo* experiments. Throughout molecular simulation using bioinformatics method, myricetin interacted with the important amino acid residues between domain 2 and 3 in SLY crystal structure, which is the binding site of SLY with cholesterol membrane. Moreover, pathogenicity of *Streptococcus suis* has depleted after myricetin treatment *in-vitro* and *in-vivo*, suggesting that SLY takes a pivotal role in the general virulence ability of *Streptococcus suis* [129]. Another natural compound, morin, has been proven to be a potent inhibitory ability towards SLY by exhibiting an interaction with SLY on its domain 2, which hindered SLY's ability to form an oligomer and leads into inhibition of SLY potentials [130].

The cholesterol binding capacity of CDCs have raised an opportunity to develop liposomal nanotraps to neutralize the cytolysins. The engineered liposomes saturated with cholesterol and containing sphingomyelin (Ch:Sm-liposomes) has proven to inhibit the hemolytic properties of recombinant PLY and SLO [127]. Furthermore, Ch:Sm liposomes are able to protect mice from *S. pneumoniae-*induced septicemia and invasive pneumococcal pneumonia [131]. Although it has not been proven in meningitis model, Ch:Sm liposomes seems to be a potential approach to combat bacterial meningitis.

## 7. Conclusions

CDCs are an important group of PFTs produced by various bacterial pathogens implicated in bacterial meningitis and have wide-ranging effects on the neurovascular unit, infiltrating leukocytes, CNS immunity and neuronal health. Given their pivotal role in brain during bacterial meningitis, CDC may be a fruitful avenue for therapeutic target of choice in meningitis over coming years.

## Use of AI tools declaration

The authors declare they have not used Artificial Intelligence (AI) tools in the creation of this article.
